# From January to June: Birth seasonality across two centuries in a rural Polish community

**DOI:** 10.1038/s41598-022-22159-3

**Published:** 2022-11-03

**Authors:** Ilona Nenko, Michael Briga, Agnieszka Micek, Grazyna Jasienska

**Affiliations:** 1 Department of Environmental Health, Faculty of Health Sciences, Jagiellonian University Medical College, Skawinska 8, 31-066 Kraków, Poland; 2 Department of Biology, University of Turku, Turku, Finland; 3 Infectious Disease Epidemiology Group, Max Planck Institute for Infection Biology, Berlin, Germany; 4 Department of Nursing Management and Epidemiological Nursing, Faculty of Health Sciences, Jagiellonian University Medical College, Kraków, Poland

**Keywords:** Biological anthropology, Social evolution

## Abstract

Seasonality of births is a worldwide phenomenon, but the mechanisms behind it remain insufficiently explored. Birth seasonality is likely to be driven by seasonal changes in women’s fecundity (i.e. ability to conceive), which is strongly influenced by their energetic status. We tested whether birth seasonality is driven by high workload and/or low access to food using 200 years of birth data, from 1782 until 2004, in an agricultural rural Polish community. First, we analysed the time series of births and within-annual variance in births, a proxy for the extent of seasonality. Secondly, we tested the hypothesis that a high agricultural workload and/or low access to food decreases number of births. We found seasonality of births throughout more than 200 years of observation in an agricultural Polish population, with a dominant birth seasonality in January and February which gradually shifted towards June in the late twentieth century. The observed pattern does not support the hypothesis that birth seasonality resulted from women’s energetic status. We discuss the possible reasons why our results do not support the tested hypothesis and some implications for our understanding of the birth seasonality.

## Introduction

Human births show seasonal variation and this is a worldwide phenomenon. Two general patterns were identified: (i) a European pattern with an excess in births during spring and summer, and a secondary peak in September and (ii) an American pattern with a trough in April–May, and a peak in September^1^. Many factors have been suggested to be related to birth seasonality, either directly or indirectly^2^, including (i) social factors such as seasonal patterns in marriages, holidays, temporal migration^3^ and the mother’s socio-demographic characteristics^4–6^, (ii) environmental factors including, for example, temperature and photoperiod^7^, and (iii) energetic factors that principally affect female fecundity^8^, including workload and food availability. Observed birth seasonality most likely results from interactions among environment, biological factors, and human volitional control^4^, all of which may vary among populations.

Identifying the mechanisms behind birth seasonality may be easier when analysing a population with a homogenous way of living relative to nationwide analyses that combine different socio-economic groups and lifestyles. In agricultural populations, seasonal workload and nutrition seem to be the most important factors affecting birth seasonality through influencing a probability of conception^2^, since ovarian function is sensitive to female energetic conditions. In the past, farm work was highly seasonal, labor-intense, and mainly done by hand, thus requiring the participation of most family members, including women. Periods of restricted energy availability, either because of low energy intake or high energy expenditure, are associated with declines in estrogen and progesterone levels, less frequent ovulations, longer cycles^2^ and consequently a lower probability of conception^9^. For example, Lese, a horticulturalist population living in Ituri Forest of the Democratic Republic of the Congo, showed a clear pattern of birth seasonality and backdated conceptions, with fewer conceptions occurring during months when women had a negative energy balance (May–July) and a higher number during the season of positive energy balance (September–November)^10^.

It has been shown previously for a Polish rural women that seasonal increases in intensity of physical activity corresponded to a reduction in ovarian progesterone levels by almost 25%^11^. High energy expenditure during harvest and haying in summer months remained in a contrast to late fall when work was less intense, and winter when women were not involved in agricultural work. Such variation has the potential to affect the seasonality of births through the suppression of ovarian hormone levels^2^. However, despite the evidence that workload suppresses ovarian function, few studies have shown that seasonal workload may also influence birth rates.

We analysed over 200 years of birth data, from 1782 until 2004, for an agricultural rural Polish community to investigate the seasonality of births and conceptions. Seasonality of birth was never before analysed in a dataset covering such a long period of time and for a population originrating from a Central European region. We analysed the data in two steps. First, we analysed the time series of births and within-annual variance in births, a proxy for the extent of seasonality. Secondly, we tested the hypothesis that high agricultural workload correlates with a decreased ability to conceive. Because the study population is agricultural with a high workload in July and August, followed by lower workload and high food abundance in September and October^11^, we expected fewer births in April and May followed by an increase in June and July.

## Results

### Temporal trends in the number, within-annual sd and within-annual cv of births

**Figure 1:**
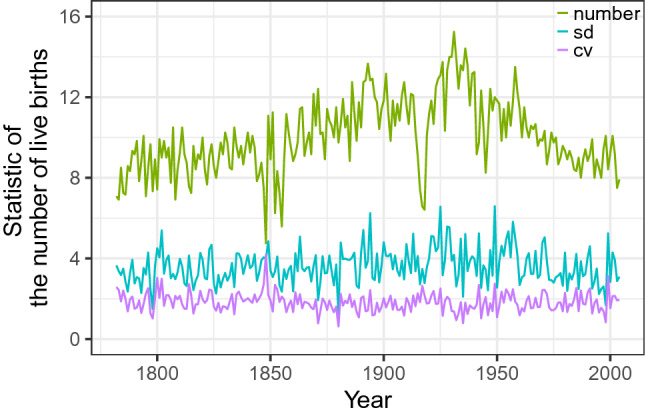
Time series showing that the monthly number of births increased with time until 1931, followed by a decline. The intra-annual standard deviation (sd) in number of births also showed a quadratic association with time, while the intra-annual coefficient of variation (cv, multiplied by a factor 5 for graphical purposes) tended to decrease with time, but both these changes were not statistically significant (Table S1).

The data contained 26,874 birth records between January 1st 1782 and December 31st 2004, with an average of 121 per year or 10 per month (annual sd = 22; 95CI = 83–164; Fig. S1A). The number of births started off at 85 per year (7 per month; Fig. 1) in 1782, increasing until a maximum of 183 per year in 1931 (15 per month; Fig. 1), and returned close to its initial value at 95 per year in 2004 (8 per month; Fig. 1). This quadratic association with time was statistically significant (ΔAICc = − 69.0; Table S1A). The size of our population was estimated at 2880 in 1827, 3869 in 1900 and 5724 in 2000, resulting in a birth rate of 36.8, 36.7 and 19.4 per 1000 inhabitants respectively. To test whether the extent of seasonality changed during the 200-year time period, we analysed whether the within-annual sd and cv changed with time, but found no evidence for any association (Fig. 1; sd: ΔAICc =  + 3.3, Table  S1B; cv: ΔAICc =  + 4.2, Table S1C) and this conclusion remained supported when redoing these analyses excluding one possibly influential data point (Cook’s D = 0.10; sd: ΔAICc =  + 0.3; cv: ΔAICc =  + 5.7). Analyses with gamms, which are more flexible than glmms with respect to inflection points, also supported this conclusion (t = 1.92, p = 0.06). Hence, contrary to our expectation, we found no support for a change in the extent of birth seasonality with time.

### The seasonality of births and successful conceptions

**Figure 2:**
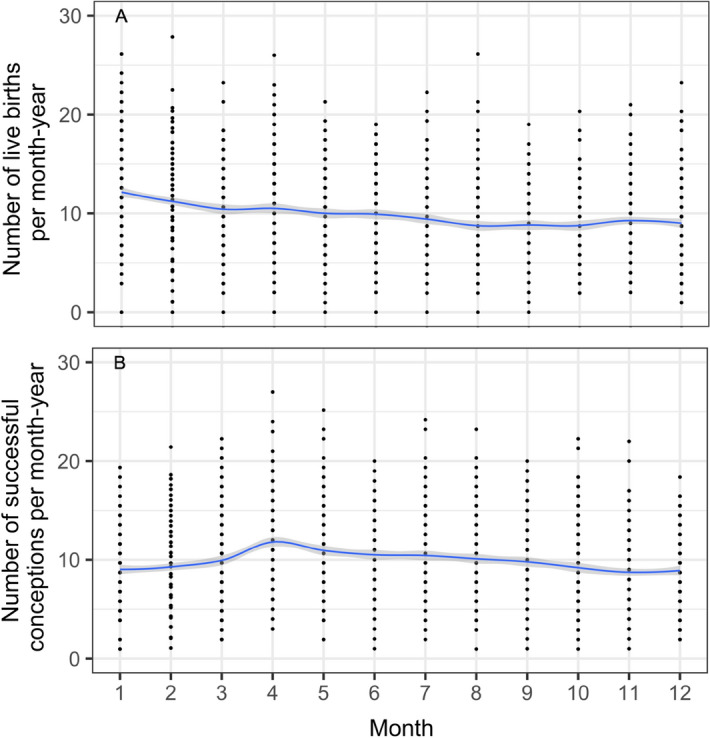
Average seasonalities of (A) births and (B) conceptions over the time period 1782-2004. Fitted lines show gamms and grey areas their 95% confidence intervals. These average seasonalities are statistically significant, as shown in Fig. S2A,B. Changes in birth seasonality after the 1950's are shown in Fig. S3 and Fig. S4A,B.

In the second aim of the study, we tested whether birth seasonality was driven by agricultural workload, which are respectively high in July and August and lower in September and October. Hence, we tested whether April and May, and June and July have respectively a low and high birth rate. On average over the 200 years of observation, we found results different from our expectations: birth maxima occurred 6 months earlier than expected i.e. in January and February and then declined gradually to a minimum from August until October (Fig. 2A). Thus, backdated successful conceptions show a maximum in April and May and a minimum from October until February (Fig. 2B). Periodograms showed that these seasonal changes were statistically significant (Fig. S2A,B). Wavelet analyses of birth seasonality over time confirmed these results, with high births in January and February for at least the first 150 years of data, but with a gradual shift towards June from the 1950’s onwards (Figs. S3, S4A,B).

## Discussion

We use an extensive dataset of over 200 years of birth data for a rural, agricultural Polish community to test the hypothesis that high workload in July and August followed by a reduction in workload in September and October drive birth seasonality. Contrary to our expectation, the peak in number of births occurred in January and February, which from the 1950s onwards gradually moved towards June. Interestingly, birth seasonality also occurred in the latter half of the twentieth century, a result confirmed by both the test of intra-annual variance in births and by wavelet analyses. Below we discuss the possible reasons why the results do not support our hypothesis and some implications for understanding the birth seasonality.

On average, during the 200 years of observation, we found a birth peak in January and February consistent with a conception peak in April and May. In the wavelet’s four early time windows (1794–1929), live births peaked early in the year, but between 1948 and 1992 this gradually moved towards April and June (Fig. S4B). A shift towards later months is consistent with results from the national data from Poland^12^ and other countries, such as Sweden in the 1850s, for which birth maxima were observed in January–March, and moved to March–April in the 1980s^13^. In Norway, no changes in birth seasonality patterns were observed between 1871 and 1996 with birth maxima always falling later relative to our study, namely in March–May^13^. The seasonal pattern in Finland was quite similar to that in Norway, with a main peak in March–April, but in addition a minor September peak^3^. Birth seasonality in Spain between 1940 and 1980s also showed a maximum in March–May^14^. In contrast, between 1876 and 1930, Switzerland showed a pattern with two maxima, one in March–May and one from October to December^15^. Thus, while many studies reported that European societies showed birth maxima in March, April or May, which is later than in the first 150 years of our study population (Fig. 2), the gradual shift towards later in the year in our population means that birth seasonality on our population tended to converge towards trends observed in other European societies.

We found no changes in the extent of birth seasonality with time (Table S1A,B), which is also different from what we expected. For example, in Spain there was a loss of birth seasonality starting from the 1990s^14^. Our study population might behave differently on that matter because it remained agricultural during the twentieth century with a minimal access to a modern contraception and a delayed experience of the demographic transition relative to other European countries. It is noteworthy, however, that some studies reported a lack of seasonality in the past, e.g. a lack of birth seasonality between 1851 and 1901 was documented for Escazú, Costa Rica^16^. Hence, while some studies reported loss of birth seasonality in the twentieth century, it is not a consistent pattern across populations and there are very few studies in which long-term changes in seasonality were analysed.

By backdating, we showed a maximum in the seasonality of successful conceptions in April–May and a minimum from October until February. It is known that human ovarian function is sensitive to energetic condition^8^, but, as outlined by Ellison et al.^8^ three particular situations should be distinguished: (i) energy status, i.e. energy stored as fat or glycogen which could be mobilized when needed, (ii) energy balance, i.e. the difference between energy input and expenditure, and (iii) energy flux, i.e. the total rate of energy turnover. Conception is more likely to occur when women are in positive energy balance and not constrained by high energy expenditure^17^. Previous research from our study population^11^ showed that energy flux of women was important for ovarian function. During high workload months—July and August—average progesterone levels were low and rose in September and October when work was less intense, which suggests that energy flux can impact ovarian function independently from energy status and energy balance. However, low sex steroid levels do not prevent conceptions, but only reduce their probability which means that women may take longer to conceive.

Another factor which may influence conceptions is photoperiod. Longer days and higher light intensity are related to a lower melatonin production which could lead to higher sex hormone levels^7^. In fact, in our study population, we observed relatively low levels of progesterone during January and February, despite the fact that women during these months had low levels of energy expenditure^18,33^. These winter months are characterised by short daylight and, in the past, very limited exposure to artificial light.

Our study, together with the previous ones, raises the question if there is an optimal time for conceptions and the ensuing births in a Central European population? One option is that a conception during spring with a birth occurring in January and February, i.e. similarly to what we found in our study, could be optimal, because it may be beneficial for both the mother and her offspring. In spring people have higher exposure to sunlight and that is necessary for the vitamin D production, which is crucial for successful reproduction, but its level is highly dependent on seasonal variation of ultraviolet B radiation (UVB)^19^. Vitamin D deficiency in pregnancy is associated with adverse maternal–fetal outcomes, e.g.: preeclampsia, gestational diabetes mellitus, preterm delivery, recurrent pregnancy loss, low birth weight, being small for gestational age, and low immunity^20^. With very limited access to (or even lack of) food such as oily fish, which contains vitamin D and lack of supplements of vitamin D in the past, humans had to rely on the vitamin D produced in the skin through exposure to sunlight.

Furthermore, with conceptions in the spring, the third trimester of pregnancy occurring during late fall/winter would provide good developmental conditions for a fetus, because this period is characterised by a relatively high food availability and low energy expenditure due to a lack of heavy agricultural work. Pregnant women with a positive energy balance could more successfully support a developing fetus during the third trimester when energy demands are high. Hence, the seasonality of conceptions and births could be driven by third trimester energy balance rather than the energy balance influencing ovarian function. Indeed, a seasonality of births that optimised infant survival probability was described in Matlab, Bangladesh where the maximum of conceptions was observed in March–April and the maximum of live births was in November, and the proportion of stillbirths was lowest in November^21^. Between 1769 and 1850 in Finland, the survival probability to one year of life was highest for children born between August–October comparing to other months of the year^22^. However, the maximum in births occurred between March and April, dropped in June–August, followed by another peak in September and hence in Finland, births did not concentrate around months with the highest infant survival. To the best of our knowledge, the reasons for the discrepancies in birth seasonality between studies remain unidentified.

Another factor that can affect birth seasonality is lactation. In the past, lactation was the best way of keeping a child alive after birth. A chance of survival of children born in winter could also increase because their mothers had more time to look after them (due that a lack of heavy agricultural work in the field). The resumption of ovarian function after lactational amenorrhea is also energy sensitive^23^. Intensive lactation in women with negative energy balance results in a longer period of ovarian suppression^24^. In our study population, traditionally women breastfed for about 12 months^25^ and this could well contribute to the seasonality of births. Resumption of menstrual cycles during winter when women had high availability of food and low energy expenditure was more probable than during other times of a year.

The observed birth seasonality in our study population could also be due to the influence of cultural factors, such as a seasonality of marriages. Marriages, especially in the past, were clustered with two peaks during the year in February and November^26^. However, while this could affect the seasonality of firstborns and in populations with low number of children per family, it is unlikely that a seasonality of first-order births drives the seasonality of all births in a population with a continuously high fertility. Data used for this study come from a population with one of the highest birth rates in Poland (18.3/1000 people vs 9.3/1000 in Poland in 2020)^27^ and hence there were few first-borns relative to their later-born siblings. A second cultural factor that can affect birth seasonality is associated with religion: the liturgical calendar prohibited sexual intercourse during Lent^28^, which can explain some of the observed changes in birth seasonality in the latter period of the time series. Even though in the early twenty-first century our study population remains very Catholic, it is well possible that the gradual secularization of society and the loosening of traditions affected or changed the birth seasonality in our population.

Our study is subject to some limitations. First, it is known that birth date falsification occurred in Poland until 1970s and children born in December were reported as being born in January of the subsequent year^12^. Hence, the birth peak in January could to some extent reflect a reporting bias. However, this bias cannot fully explain the long-lasting birth peak until February and the gradual decline in birth rate towards autumn (Fig. 2A). 

A second limitation of our data is that they are based on live births and successful conceptions only. We have calculated a month of conception by subtracting 40 weeks from the month of birth following procedure used in previous studies on seasonality^10,29^. A 9-month time period is used to estimate conceptions from births^4,10,30^. However, it is possible that not every pregnancy was 40 weeks or 9 months long and shorter or longer pregnancies also occurred. Information about the accurate duration of pregnancy is impossible to obtain for historical populations when data originate from parish records or national censuses. Additionally, abortions and stillbirths were not recorded. Warren and colleagues^31^ found no seasonal variation in spontaneous nor induced abortion. In a recently published study, 20% of women experienced spontaneous abortions (a loss of pregnancy before 20 weeks’ gestation) and the highest risk for spontaneous abortion was observed in late August^32^, which would cause a birth minimum in December, a pattern which is also not consistent with our results (Fig. 2). However, it should be noted that a vast majority—two thirds—of spontaneous abortions occur before pregnancies are clinically recognized^2^.

A third limitation is that sample size can limit the ability to detect seasonality. This might explain some of the statistically non-significant periods in the wavelet analyses (Fig. S4A). For example, while it remains difficult to explain why wavelet analyses showed a time window with statistically significant birth seasonality and other without, there is a window between 1947 and 1952, which coincides with the brief increase in births just after WWII (Fig. 1). However, we think it is unlikely that the sample size drives the dynamics of birth seasonality because if there were, (i) the seasonality would match the sample sizes as shown in Fig. 1 (green line), which is not the case (Figs. 2A, S3) and (ii) our study population dates back from a period with high birth rates at > 36 per 1000 inhabitants and remains a population with one of the highest birth rates in contemporary Poland.

To conclude, we tested the hypothesis that the seasonality of live births and successful conceptions in an agricultural Polish population during > 200 years could be driven by the seasonal dynamics of agricultural workload and food abundance through the influence of these energetic factors on ovarian function. Instead, we found a very different seasonal dynamic than expected from this hypothesis and relative to other European countries. Our study indicates that the seasonality of conceptions and subsequent births is likely influenced by various biological, environmental and cultural factors, and their interactions.

## Methods

### Study population and data collection

We used demographic data from parish records in the village of Slopnice in southern Poland, belonging to the area of the Mogielica Human Ecology Study Site^33,34^. The population has been catholic and vicars were obliged by the Council of Trent in 1563 to record all births, marriages, and deaths^35^. The Slopnice population was characterized by a very high birth rate and it still has one of the highest in Poland^27^. Until recently, this population was characterized by natural fertility, due to strongly rooted religious beliefs, as well as a lack of modern contraceptive methods, but it is currently experiencing a demographic transition^36^.

Agricultural life has been characterized by seasonality, including a seasonal access to food. The diet of rural people was very simple, based on potatoes, cabbage, beans, milk (with exception of winters when cows were calving), rye, barley, buckwheat groats and beetroots^37^. Dietary shortages occurred especially during the so called ‘hungry gap’ lasting from spring until a new harvest^38^. Fields were located on slopes of mountains and mechanizations was rarely used. In these conditions, women’s involvement in agricultural work was very high. People lived in small wooden houses with a lack of electricity and running water. Stilled stoves were started to be built after World War II. During the time of communism (from 1945 to 1989), Polish farmers had to rely mostly on products which they were able to produce themselves^39^, because access to food products in shops in villages was limited. Significant changes in the way of life were observed during last 25 years of XX century^37^. All methods were carried out in accordance with relevant guidelines and regulations. The study was approved by the Jagiellonian University Bioethical Committee.

### Statistical analysis

We performed the analyses with the number of live births and successful conceptions using complete years between January 1st 1782 and December 31st 2004 (Fig. 1). To estimate the date of successful conceptions, we subtracted 40 weeks from the birth date. The subtraction of 40 weeks ignores variation in the length of pregnancies, but this variation is within the monthly resolution of the aforementioned hypotheses and analyses (see wavelet analyses below). We standardized the numbers of births and conceptions per month to 30 days to take into account differences in the length of the month.

### Temporal trends in the number and within-annual variation of births

In this study, we performed two analyses. In the first analysis, we described (i) the temporal trends in the time series of the number of births, (ii) their within-annual standard deviation (sd) and (iii) their within-annual coefficient of variation (cv, cv = within-annual sd/annual mean). Both, the sd and cv reflect within-annual change, and hence are a proxy for seasonality, thereby allowing to test for changes in the extent of seasonality with time, but the cv has the advantage that it corrects for temporal trends in the number of births, which can be important because the sd often increases with the mean.

For these analyses, we followed the general linear modelling approach, as explained in^40^ using the function *‘gls’* of the package *‘nlme*’^41^ in R^42^. In these models, to determine the statistical ‘significance’ of predictor variables, we used model selection based on the second order Akaike Information Criterion (AICc): better fitting models are indicated by their lower AICc values, with models within 4 ΔAICc being plausible and becoming increasingly equivocal up to 14 ΔAICc, after which they become implausible^43,44^.

We estimated the within-annual sd and cv using monthly time intervals. In all models, the predictor variables were year and year^2^, standard normalized to a mean of 0 and a sd of 1. We checked robustness of the findings by repeating all analyses fitting the more flexible general additive models (gamms), allowing to explore non-linear shapes of the associations. To this end, we used the function *‘gamm’* of the package *‘mgcv’* Woods, S. 2017. Generalized Additive Models. An Introduction with R (Second Ed.). Chapman & Hall, Boca Raton, Fl. (see Fig. S4B)

All data followed a normal distribution (Fig. S1A–C) and were log-transformed for the analyses. We corrected for temporal autocorrelation by including year as an auto-regressive factor of order 1^45^, although models with or without temporal autocorrelation fitted almost equally well (births: ΔAICc =  + 1.9; sd: ΔAICc =  + 2.1; cv: ΔAICc =  + 2.1). In all models, we accounted for possible heteroscedasticity with the ‘weights’ function, which improved the model fits for births (births: ΔAICc = − 6.3; sd: ΔAICc =  + 1.7; cv: ΔAICc =  + 1.7). We visually inspected model residuals for other assumptions, e.g. Q-Q plots^45^, and checked for influential datapoints with the function *‘CookD’* from the package *‘predictimeans’*^46^.

### The seasonality of births and successful conceptions

In the second analyses, we identify whether births showed recurring seasonality using wavelet analyses. Wavelet analyses are an approach used in many fields, including ecology, demography, epidemiology, economics and engineering, to detect trends in non-stationary time series, i.e., systems which properties change through time^47,48^. In brief, wavelet analysis decomposes time series data using functions (wavelets) simultaneously as a function of both period and time^48^. Because the time component is continuous, the approach requires no assumptions regarding when changes occur or arbitrary compartmentalizing of the time series. Hence, this approach is useful, for example, to detect whether the seasonality changes over time^47,48^.

For the wavelet analyses, we fitted a Morlet wavelet in R^42^ using the function ‘analyse.wavelet’ from the package ‘WaveletComp’^49^ and quantified a periodicity between 6 months and 2 years, with 1 year indicating an annually recurring seasonal signal. Performing the analyses using weekly or monthly intervals gave consistent conclusions (results not shown) and for the ease of interpretation we present the monthly analyses. To avoid the results being driven by local peaks, we smoothened and detrended time series following standard procedures^49^. To determine statistical significance, we then compared the data’s periodicity with that of 1000 ‘white noise’ datasets with significance at the 5% level. Once we detected a statistically significant seasonal signal, we identified the month(s) of peak births based on the monthly counts.

## Supplementary Information

Supplementary Information.

## Data Availability

The datasets analysed during the current study are available from the corresponding author on reasonable request.
