# Author Correction: From January to June: Birth seasonality across two centuries in a rural Polish community

**DOI:** 10.1038/s41598-022-25709-x

**Published:** 2022-12-06

**Authors:** Ilona Nenko, Michael Briga, Agnieszka Micek, Grazyna Jasienska

**Affiliations:** 1 Department of Environmental Health, Faculty of Health Sciences, Jagiellonian University Medical College, Skawinska 8, 31-066 Kraków, Poland; 2 Department of Biology, University of Turku, Turku, Finland; 3 Infectious Disease Epidemiology Group, Max Planck Institute for Infection Biology, Berlin, Germany; 4 Department of Nursing Management and Epidemiological Nursing, Faculty of Health Sciences, Jagiellonian University Medical College, Kraków, Poland

Correction to: *Scientific Reports* 10.1038/s41598-022-22159-3, published online 03 November 2022

The Funding section in the original version of this Article was incomplete.

"This work was supported by the National Science Centre [grant number 2016/21/D/NZ8/01306 (IN)]; the Ministry of Science and Higher Education [grant number IdP2011000161 (GJ)]; and the Ella & Georg Ehrnrooth Foundation (MB)."

now reads:

“This work was supported by the National Science Centre [grant number 2016/21/D/NZ8/01306 (IN)]; the Ministry of Science and Higher Education [grant number IdP2011000161 (GJ)]; and the Ella & Georg Ehrnrooth Foundation (MB). Publication of this article was funded by the Priority Research Area qLife under the program “Excellence Initiative – Research University” at the Jagiellonian University in Krakow (06/IDUB/2019/94).”

The original Article has been corrected.

