# Supplementary material for: From January to June: Birth seasonality across two centuries in a rural Polish community
Source: Sci Rep. 2022 Nov 3;12:18579. doi: 10.1038/s41598-022-22159-3 (PMC9633606; doi:10.1038/s41598-022-22159-3)

**Supplementary information to:**

**From January to June:**

**Birth seasonality across two centuries in a rural Polish community**

Ilona Nenko^1^, Michael Briga^2,3^, Agnieszka Micek^4^, Grazyna Jasienska^1^

^1^Department of Environmental Health, Faculty of Health Sciences, Jagiellonian University Medical College, Krakow, Poland

^2^Department of Biology, University of Turku, Turku, Finland

^3^Infectious Disease Epidemiology Group, Max Planck Institute for Infection Biology, Berlin, Germany

^4^Department of Nursing Management and Epidemiological Nursing, Faculty of Health Sciences, Jagiellonian University Medical College, Krakow, Poland

Table S1. Model selection tables showing the model support for a quadratic association with time for (A) number of successful births, but not for (B) the within-year standard deviation (sd), nor for (C) the within-year coefficient of variation (cv).

|  | **Coefficients** |  |  | **Model selection** | |  |
| --- | --- | --- | --- | --- | --- | --- |
| **Intercept** | **year** | **year2** | **df** | **AICc dAICc** | | **weight** |
| **(A) Number of births** | | | | | | |
| 4.87 | 0.05 | -0.09 | 6 | -181.1 | 0.0 | 1.0 |
| 4.78 | 0.08 |  | 5 | -123.5 | 69.0 | 0.0 |
| 4.78 |  |  | 4 | -104.3 | 113.0 | 0.0 |
| **(B) Within-year standard deviation** | | | | | | |
| 1.25 |  |  | 4 | 29.3 | 0.0 | 0.7 |
| 1.31 | 0.02 | -0.06 | 6 | 32.6 | 3.3 | 0.1 |
| 1.25 | 0.03 |  | 5 | 32.6 | 3.3 | 0.1 |
| **(C) Within-year coefficient of variation** | | | | | | |
| -1.04 |  |  | 4 | 50.3 | 0.0 | 0.9 |
| -1.04 | -0.04 |  | 5 | 54.5 | 4.2 | 0.1 |
| -1.07 | -0.03 | 0.04 | 6 | 59.0 | 8.7 | 0.0 |

Figure S1. Distribution of the annual number of births, the intra-annual sd and intra-annual cv.

Figure S2. Statistical support for a recurring seasonal pattern across the time series between 1782 and 2004 for (A) live births and (B) successful conceptions. Horizontal black line shows to periodicity of 1 year, i.e. a seasonal recurring pattern. Red and blue dots show statistical significance at respectively .05 and .1, based on the simulation of 1,000 random datasets. The differences in wavelet power between both variables are minor, e.g. Cazelles et al. 2007.


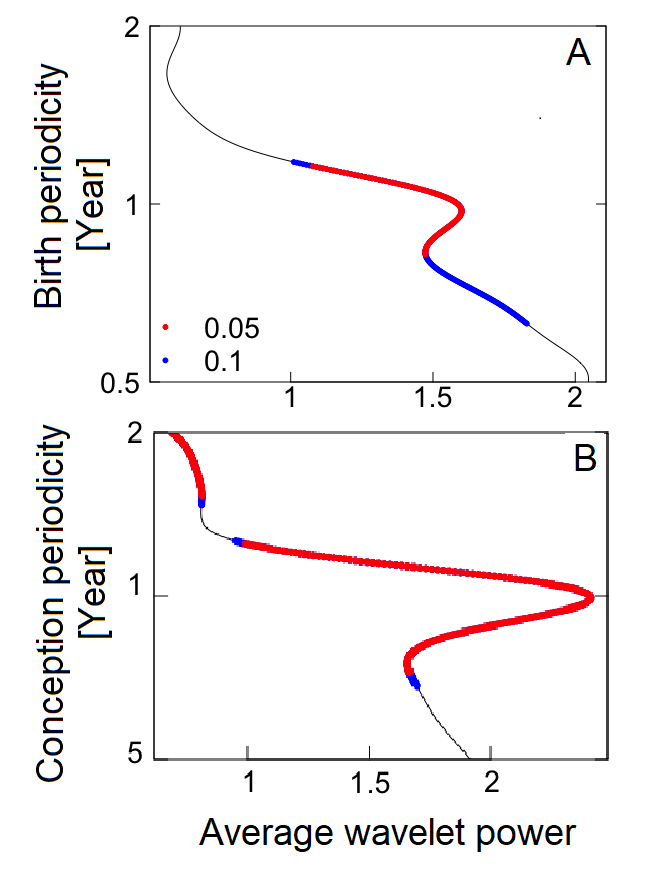


Figure S3. Birth seasonality across the time series 1782-2004, showing a dominant seasonality in January and February in the time series until the 1950’s, which was followed by a gradual shift towards summer. Statistical analyzes of these time series are shown in Fig S4.


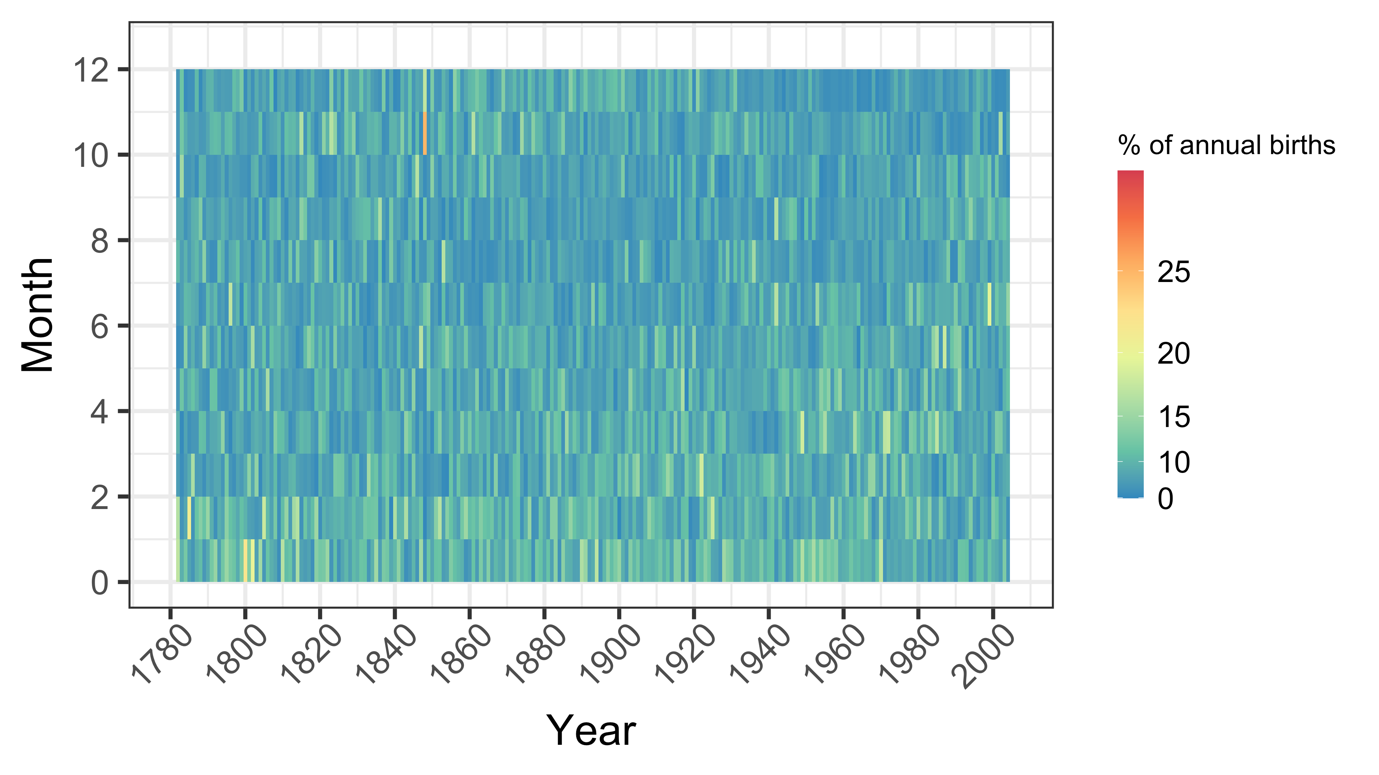


Figure S4. (A) Results of wavelet analyzes with seven squares zooming in on the time windows with statistically significant annual recurring birth seasonality (1832-1835, 1860-1865, 1881-1884, 1910-1914, 1924-1928, 1954-1957, 1989-1993). (B) Zoom of time windows, illustrating the dominant birth seasonality in January and until the last two windows when births occurred more in June. Fitted lines show general additive models (gamms) following Woods 2017 and grey areas represent 95% confidence intervals.


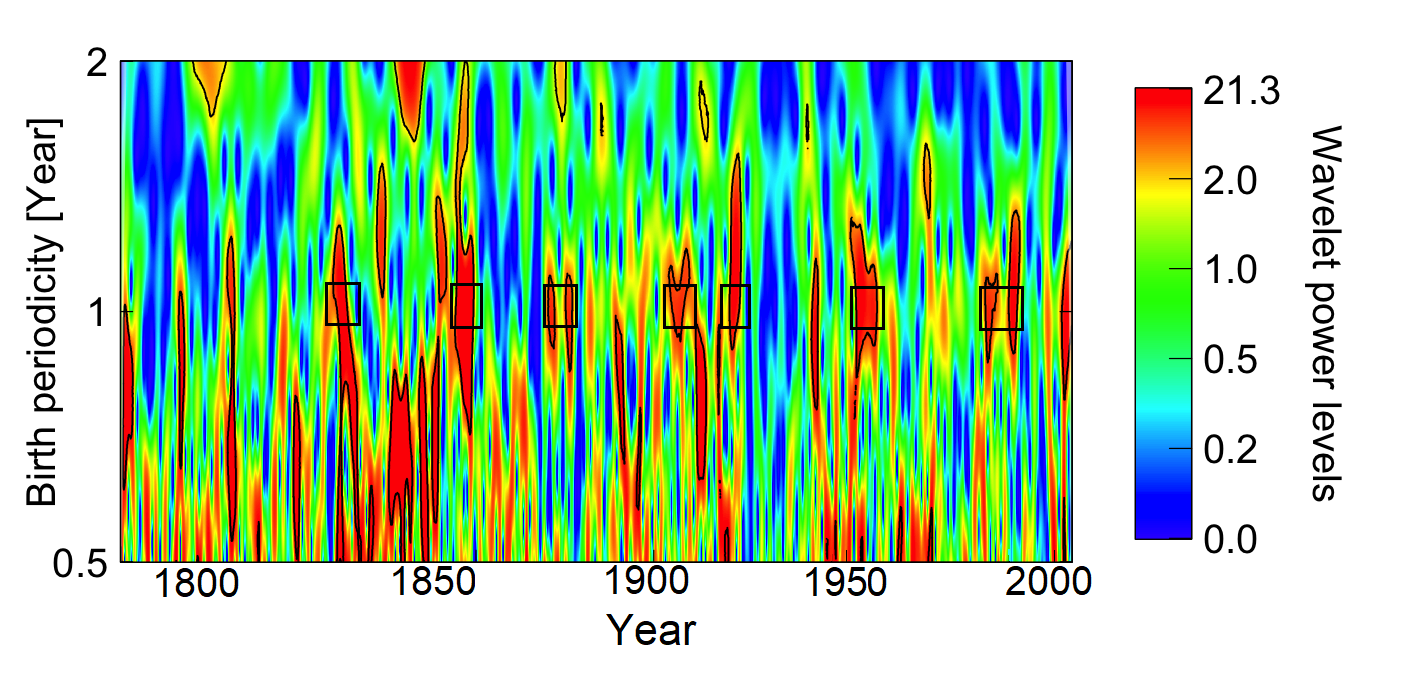


A

B


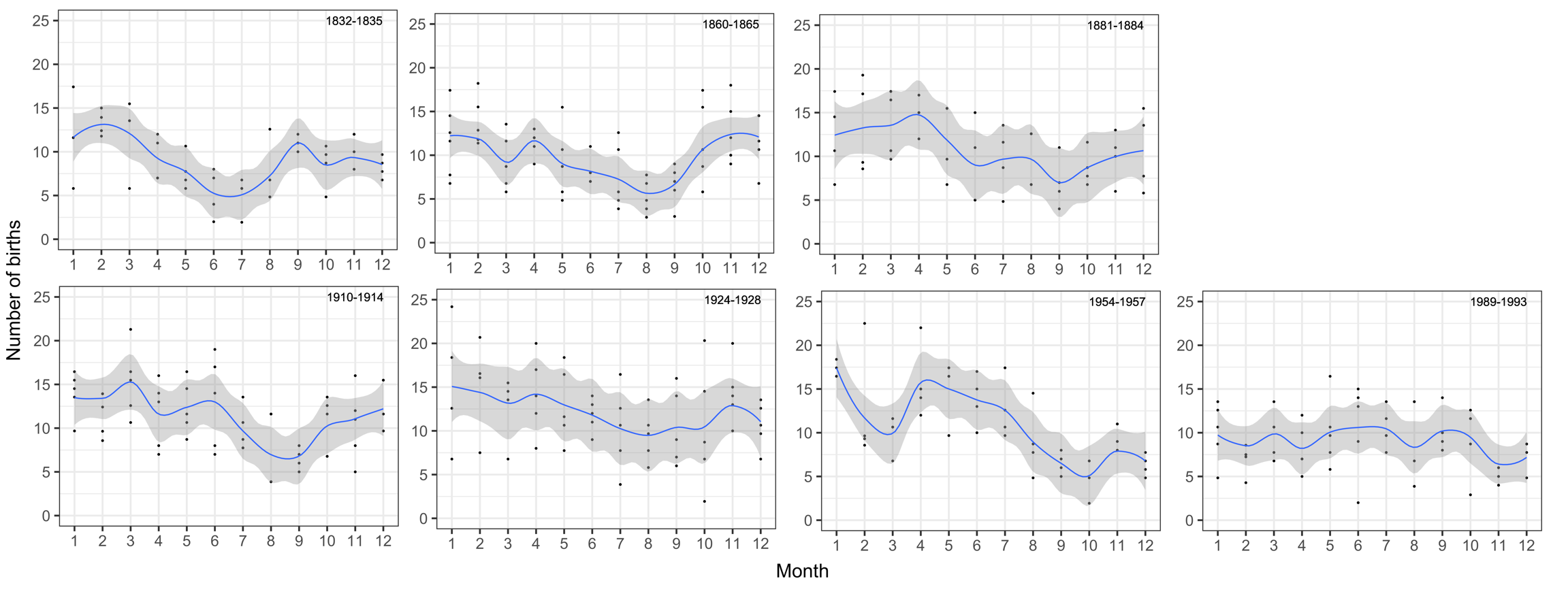

Supplement: Supplementary file 1 — Supplementary Information. [file 41598_2022_22159_MOESM1_ESM.docx]
